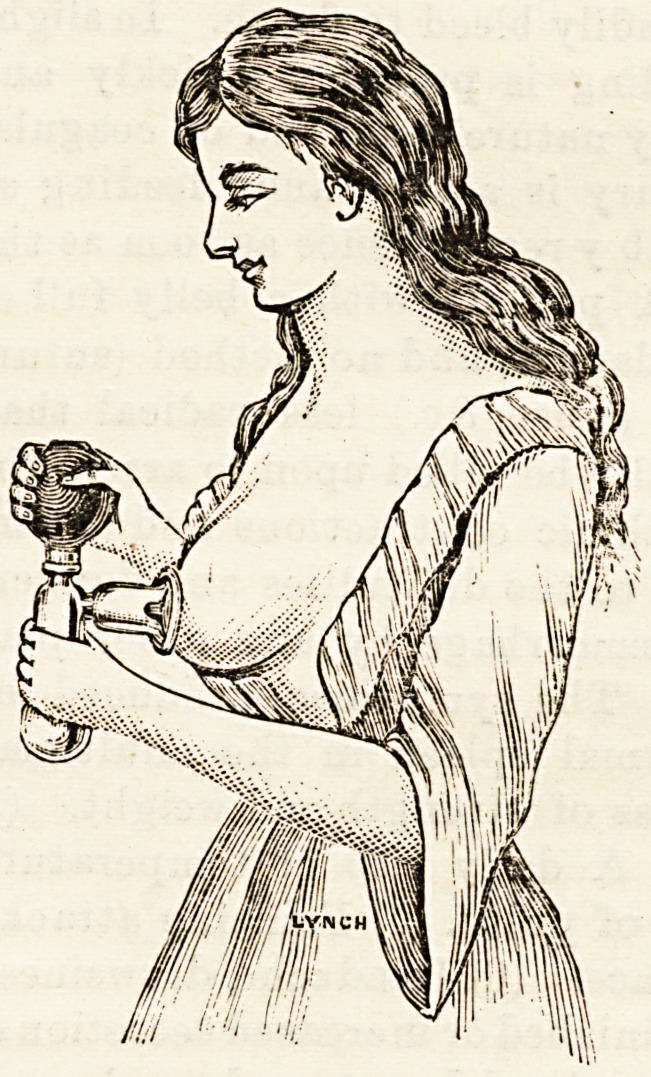# New Appliances and Things Medical

**Published:** 1898-11-05

**Authors:** 


### NEW APPLIANCES AND THINGS MEDICAL.

[We shall be glad to receive, at our Office 28 & 29, Southampton Street, Strand, London, W.C., from the manufacturers, specimens of all new preparations and appliances which may be brought out from time to time.]

### NEW BREAST RELIEVER.

(Lynch and Co., Limited, 192, Aldersgate Street, London, E.C.)

This is a very good breast pump, and possesses features which are not always to be met with in instruments of its class. The accompanying sketch shows the shape of the apparatus. It will be observed that the glass reservoir into which the milk flows forms at the same time a convenient handle by which the shield can be held comfortably against the nipple, and that from the relative positions of the reservoir and pump when so held the abstraction of the milk can be performed without that movement of and pressure upon the swollen and often tender breast which is almost inevitable when the ball of the pump is placed in a line with the nipple shield, instead of, as in this case, transversely to it. The pump is made of good rubber, and thus the apparatus is not only effective but is easily regulated in regard to the force with which it acts upon the breast. Among its other advantages attention may be drawn to the size of the reservoir, for in the use of a breast reliever it is distinctly desirable not to have to "break off" to empty the bulb.

## Figures and Tables

**Figure f1:**